# Foliar application of putrescine alleviates terminal drought stress by modulating water status, membrane stability, and yield- related traits in wheat (*Triticum aestivum* L.)

**DOI:** 10.3389/fpls.2022.1000877

**Published:** 2023-04-21

**Authors:** Allah Wasaya, Iqra Rehman, Atta Mohi Ud Din, Muhammad Hayder Bin Khalid, Tauqeer Ahmad Yasir, Muhammad Mansoor Javaid, Mohamed El-Hefnawy, Marian Brestic, Md Atikur Rahman, Ayman El Sabagh

**Affiliations:** ^1^ Department of Agronomy, Bahauddin Zakariya University Multan, Multan, Pakistan; ^2^ College of Agriculture, University of Layyah, Layyah, Pakistan; ^3^ National Research Center of Intercropping, The Islamia University of Bahawalpur, Multan, Pakistan; ^4^ Department of Agronomy, College of Agriculture, University of Sargodha, Sargodha, Pakistan; ^5^ Department of Chemistry, Rabigh College of Sciences and Arts, King Abdulaziz University, Jeddah, Saudi Arabia; ^6^ Department of Plant Physiology, Slovak University of Agriculture, Nitra, Slovakia; ^7^ Grassland and Forage Division, National Institute of Animal Science, Rural Development Administration, Cheonan, Republic of Korea; ^8^ Department of Agronomy, Faculty of Agriculture, Kafrelsheikh University, Kafr al-Sheik, Egypt; ^9^ Department of Field Crops, Faculty of Agriculture, Siirt University, Siirt, Türkiye

**Keywords:** bread wheat, yield, terminal drought, putrescine, leaf area ratio, membrane stability index

## Abstract

Drought stress is one of the major limitations to the growth and yield productivity of cereal crops. It severely impairs the early growing and grain -filling stages of wheat. Therefore, cost- effective and eco-friendly approaches for alleviating drought stress in cereal crops are in high demand. Polyamines, such as putrescine, have a significant effect on improving crop yield under drought- stress conditions. Therefore, the current study was executed with the aim of exploring the significance of putrescine in alleviating drought stress and improving yield- related traits in wheat. Two distinct wheat cultivars (Fakhar-e-Bhakkar and Anaj-2017) were treated with the foliar application of different concentrations (control, 0.5, 1.0, and 1.5 PPM) of putrescine (put) under two moisture conditions (well- watered and terminal drought stress). The results demonstrate that the imposition of terminal drought stress significantly reduces different physiological and yield- related traits of both wheat cultivars. The reduction of relative water content (RWC%), membrane stability index (MSI), leaf area, tillers per plant, biomass yield, number of spikelets per spike, 100-grain weight, grain yield per plant, and straw yield was greater in Anaj-2017 than in Fakhar-e-Bhakkar cultivar. The results further explain that the foliar application of increased concentrations of putrescine from 0.0 to 1.0 PPM gradually improved physiological and yield traits, whereas these traits declined with the application of putrescine at the highest dose (1.5 PPM). The exogenous application of 1.0 PPM putrescine improved the relative water content (19.76%), specific leaf area (41.47%), and leaf area ratio (35.84%) compared with the controlled treatment. A higher grain yield (28.0 g plant^-1^) and 100-grain weight (3.8 g) were obtained with the foliar application of 1.0 PPM putrescine compared with controlled treatments. The findings of this study confirm the protective role of putrescine against terminal drought stress. It is therefore recommended to use putrescine at a concentration of 1.0 PPM, which could help alleviate terminal drought stress and attain better wheat yield.

## Introduction

Drought stress is a serious threat to cereal crop growth, development, and productivity (Toulotte et al., 2022). The early growth and grain- filling phases of cereal crops are significantly affected by drought (Vijayaraghavareddy et al., 2021). Low water levels reduce vegetative growth, post-anthesis photosynthetic capacity, and grain yield in wheat (Cui et al., 2015). Drought occurs frequently in fields that alter the physiological and molecular characteristics of plants (Rahman et al., 2022; Islam et al., 2023 ). Drought stress reduces cell division, cell proliferation, and stem elongation, which leads to severe impairments to plant growth and productivity (Azooz and Youssef, 2010; Farooq et al., 2013; Wasaya et al., 2018). Plant morphology and physiology are adversely affected by environmental stresses, including drought, which ultimately reduce crop growth and agricultural yield (Sabagh et al., 2020). Drought stress leads to regulating several oxidative stress indicators, including the generation of reactive oxygen species (ROS), cellular injury, peroxidation of lipids, and membrane stability in plants (Kar, 2011; Rahman et al., 2015; Sabagh et al., 2021). Therefore, affordable and eco-friendly approaches are highly desirable for the improvement of economically important cereal crops under drought- stress conditions. Several eco-friendly approaches have been documented for abiotic stress tolerance and plant improvements (Sabagh et al., 2021; Khan et al., 2022; Raza et al., 2022a; Raza et al., 2022b). A numerical group of signaling molecules and growth regulators has been reported to alleviate drought stress in economically important crop plants, including cereals (Abd Elbar et al., 2019; Rahman et al., 2021; Islam et  al.,2022).

Wheat (*Triticum aestivum*) is one of the most widely cultivated cereal crops across the globe, from subtropical to temperate climates, predominantly in the Mediterranean and semi-arid regions (Ahmed et al., 2019). It directly contributes to food security as it is considered the second-most significant staple food crop in the world (Zaheer et al., 2021). However, global plant production is at risk due to environmental changes, a decline in water levels, and irregular rain patterns, which severely influence crop growth stages (Nielsen et al., 2010; Rahman et al., 2018; Schmidt et al., 2020; Ahmed et al., 2022; Chauhan et al., 2022). For instance, the flowering and grain- filling stages of wheat are highly critical for yield protection, whereas the onset of terminal drought stress during these phases may cause a reduction in the number of kernels/ears and kernel weight and may be responsible for huge yield loss (Liu et al., 2015; Liu et al., 2016; Ebeed et al., 2017; Dong et al., 2017; Habib-ur-Rahman et al., 2022). Similarly, drought stress at anthesis can inflict a more profound impact on grain filling, leading to a shortened period of grain filling and altered enzymatic activities (Ahmadi and Baker, 2001; Shah and Paulsen, 2003; Pireivatlou, 2010; Farooq et al., 2014). Drought causes tissue dehydration, leading to metabolic impairment at critical growth stages of wheat crops (Farooq et al., 2009b; Hasan et al., 2021). Moreover, terminal drought disturbs stomatal oscillations, reduces transpiration and water- use efficiency (WUE), and decreases chlorophyll content and nutrient uptake, collectively resulting in yield reduction (Farooq et al., 2009a; Dawood et al., 2019; Yasir et al., 2019). It also imposes adverse effects on the physio-morphological responses of plants, resulting in significant yield reduction (Iqbal et al., 2019; Ejaz et al., 2022). For instance, drought stress induces the excessive production of ROS and inhibits antioxidant enzyme activity, which impairs photosynthesis, increases lipid peroxidation, and destroys cell membrane structure (Farooq et al., 2009a; Miller et al., 2010; Ju et al., 2020). Since crop genotype is an important determinant of drought response, different cultivars within crop species may differ strongly in their response and adaptation to drought stress (Abid et al., 2018; Mohammadi et al., 2018). Therefore, the response of wheat genotypes against drought stress, combined with their stress mitigation potential, must be studied to improve the drought tolerance of wheat genotypes in Pakistan.

Nowadays, different strategies are being employed to mitigate drought stress in crop plants. One such strategy is the exogenous application of polyamines (PAs) (Phornvillay et al., 2019; Seifikalhor et al., 2020; Zhang et al., 2020; Çığ et al., 2021; Irshad et al., 2021). PAs, such as putrescine, are responsible for accumulating osmolytes and endogenous PAs in stressful environments, which help crop plants to protect themselves against stressful conditions (Ebeed et al., 2017). Putrescine (Put) is a type of PA found in all living organisms (Abd Elbar et al., 2019). The exogenous application of putrescine alleviated drought stress by regulating physio-biochemical traits in sugar beet (Islam et  al.,2022), regulating protein and fatty acids in thylakoid membrane under several abiotic stresses, including drought in plants (Rahman et al., 2014; Shu et al., 2015), and improving shoot/root dry matter ratio, morph-anatomical changes in roots, stems, and leaves to avoid tissue dehydration in *Thymus vulgaris* (Abd Elbar et al., 2019). Additionally, putrescine helps enhance the stability of cell membranes by preventing excessive water loss, ultimately retaining photosynthetic efficiency under adverse environmental conditions (Grigorova et al., 2012). The foliar spray of putrescine can trigger different physiological processes and induce osmotic adjustment in plants (Chen et al., 2019). It was also reported that putrescine foliar spray might slow aging and protect cell membranes from oxidative damage by removing free radicals in plants under drought-stress conditions (Hussein et al., 2019). These studies justify the beneficial effect of Put in plant abiotic stress. Under the current climate change scenario, there is a high demand for eco-friendly strategies for abiotic stress alleviation in crop plants in arid and semi-arid regions. Therefore, this study was undertaken to explore the insights of Put-mediated drought stress alleviation in wheat, along with how Put regulates agricultural and physiological yield attributes under water- deficient conditions. These studies open a new avenue of drought- stress alleviation in field crop research that might be useful to the plant breeder and farmer for improving drought stress tolerance in plants through breeding programs.

## Materials and methods

### Experimental sites, plant material, and treatments

The present experiment was conducted under Lath House at the College of Agriculture, Bahauddin Zakariya University, Bahadur Sub-Campus Layyah, Punjab, Pakistan. Viable seeds of two different wheat varieties, Fakhar-e-Bhakkar (V1) and Anaj-2017 (V2), arranged by the Arid Zone Research Institute (AZRI) Bhakkar, Punjab, Pakistan, were used as experimental material. The pots were filled with soil and farmyard manure at a ratio of 2:1. Earthen pots (14 cm in diameter and 70 cm in height) were filled with 11 kg of well- pulverized sieved soil. The pots were irrigated uniformly and then, upon reaching optimum moisture conditions, eight seeds per pot were sown at a depth of 2 cm on 20^th^ November 2020. The pots were irrigated regularly, and thinning was performed at the three-leaf stage of the seedlings to keep three plants per pot for observations. Commercial fertilizers were applied according to the recommended doses as follows: 0.66 g pot^-1^ nitrogen (as urea), 0.55 g pot^-1^ phosphorus as DAP (di-ammonium phosphate), 0.44 g pot^-1^ potassium as SOP (sulfate of potash) respectively. The crop was grown until the heading stage without drought imposition by watering the pots as and when required. At the heading stage, the pots were divided into two groups: well-watered (normal or control) and terminal drought (40% water holding capacity (WHC)). To maintain WHC, the pots were weighed regularly after every alternative day of drought imposition, and a difference in weight was achieved by adding water to attain the required weight. After one week of drought imposition at the heading stage, the wheat plants were sprayed with four different concentrations (PPM) of putrescine *viz.* putrescine 0 (control, put1), 0.5 (put 2), 1.0 (put 3), and 1.5 PPM (put 4). In the control treatment, plants were sprayed with distilled water, while the rest of the treatment groups were sprayed with different concentrations of putrescine, as per the treatment doses. The experiment containing three replications of each treatment under the factorial arrangement was laid out as a Completely Randomized Design (CRD). The experiment consisted of 48 experimental pots in total. Wheat plants were harvested on 14^th^ April 2021 at physiological maturity. The harvested plants were sundried for a week and yield-related traits were collected.

### Measurement of leaf area, specific leaf area, and leaf area ratio

Leaf area (LA), specific leaf area (SLA), and leaf area ratio (LAR) were determined after 7 days of putrescine treatment. The LA, SLA, and LAR were calculated from destructive flag leaf samples by following a distinct formula. The following Quarrie and Jones LA equation was used for measuring leaf area (Aldesuquy et al., 2014).


$$\text{Leaf area (}cm^{2})=\text{Length }\times \text{Width}\times 0.75$$

The SLA and LAR were calculated using following formula (Amanullah et al., 2007).


$$\text{Specific Leaf Area}=\text{Leaf area}÷{\text{leaf dry weight plant }}^{-1}(cm^{2}g^{-1})$$

$$Leaf\;Area\;Ratio=Leaf\;area\;plant^{-1}÷total\;dry\;weight\;plant{\;}^{-1}(cm^{2}g^{-1})$$

The plant height (cm), number of tillers per plant, and plant biomass were measured for non-treated and treated plants. Plant samples were kept at 71°C in a hot air oven for 48 hours, then total biomass yields (dry weight basis) were recorded for two distinct wheat varieties, respectively.

### Determination of membrane stability index

After 7 days of foliar application of putrescine, fully matured leaves were detached from each treated plant. Two pieces of fresh flag leaves, each of 0.2 g, were taken and divided into small strips then put in glass test tubes containing 10 ml of distilled water. Two sets of test tubes were prepared. One set of test tubes was put in a water bath for approximately 30 minutes with the temperature set at 40°C, then the EC (electrical conductivity) of each test tube sample was recorded using an EC meter and was designated C_1_. Meanwhile, the second set of test tubes was put in a water bath for about 15 minutes at 100°C; its EC (electrical conductivity) was noted and represented as C_2_. The MSI was estimated according to the given formula presented by Sairam et al. (1997).


$$\text{MSI}=\left[1-\left(\frac{{\text{C}}_{1}}{{\text{C}}_{2}}\right)\right]\times 100$$

### Determination of relative water content

Fresh, fully emerged flag leaves were collected from each experimental pot after 7 days of putrescine application and kept in a polythene bag, which was quickly shifted in the laboratory to estimate the relative water content (RWC). After moving the plant samples to the laboratory, the fresh weight (FW) of the detached leaves was recorded. Afterward, these leaves were dipped in distilled water and kept in dark conditions at room temperature for 24 hrs to record the turgid weight (TW). After recording the TW, leaf samples were air -dried in a hot air oven at a temperature of 70°C. The leaf samples were completely dried until a constant weight was achieved, then the dry weight (DW) was recorded using an electronic digital balance. The RWC was estimated according to the formula presented by Barrs and Weatherley (1962).


RWC=[FW−DW/TW−DW]×100

### Determination of yield -related traits

Yield and related traits, like spike length (cm) per plant, spike weight (g) per plant, number of spikelets per spike, 100-grain weight (g), grain yield per plant, harvest index (HI), and straw yield were recorded for the wheat varieties, respectively, after plant harvesting. Three plants from each pot were randomly selected and the productive tillers per plant were counted manually. Moreover, the height (from the stem base to the apex of the ear) of three plants at maturity was recorded *via* a meter scale and was later averaged. Similarly, ten spikes per pot were selected randomly, and the length of each spike was measured using a scale then averaged to determine the spike length per plant. T hese selected spikes were then used for the calculation of the number of spikelets per spike and averaged. Three plants were harvested manually after reaching physiological maturity and sundried for a week, and biological yield was recorded using an electrical balance. The spikes were then separated from these three plants and threshed to record grain yield and the number of grains per spike. After threshing, 100 grains were counted from the seed lot of each pot to record the 100-grain weight. The harvest index (HI) was calculated considering the ratio of grain and biological yield as a percentage (%).

### Statistical analysis

Data regarding all the traits were analyzed statistically by performing an analysis of a variance (ANOVA) test using the software, Statistix 8.1. Tukey’s test was applied in order to draw data means and the significant difference at P<0.05 (Steel et al., 1997). Bar graphs for the mean and interactive study were generated on graphing and analysis software, OriginPro Version 9.8.0.200.

## Results

### Effect of putrescine on the leaf area of wheat genotypes under drought stress

**Figure 1:**
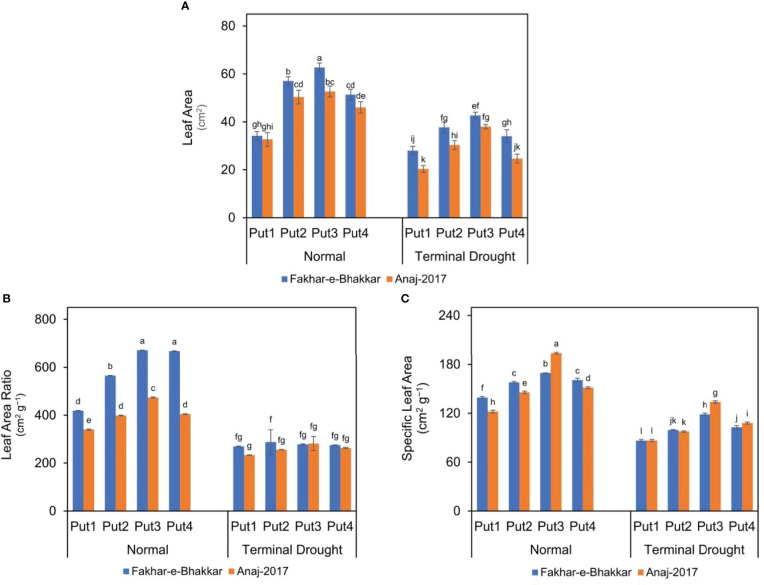
Effect of putrescine on the leaf area **(A)**, leaf area ratio **(B)**, and specific leaf area **(C)** of two wheat cultivars under normal and terminal drought conditions. Abbreviation: PPM, parts per million, Put1, putrescine 0 PPM (untreated control); Put2, putrescine 0.5 PPM; Put3, putrescine 1.0 PPM; Put4, putrescine 1.5 PPM. The different letters above the bar columns indicate significant differences in mean ± SE at p< 0.05. At least three independent biological replications were considered for each treatment.

Drought stress significantly reduced the LA, LAR, and SLA of both wheat genotypes. Moreover, these indices were considerably reduced in the Anaj-2017 (V2) wheat variety compared to the Fakhar-e-Bhakkar (V1) variety (**Figure 1**). However, the putrescine application mitigated the impact of drought stress in both the genotypes, and the foliar application of 1.0 PPM seemed most suitable against drought stress. Maximum LA and LAR were recorded in Fakhar-e-Bhakkar with the foliar application of 1.0 PPM putrescine, while the minimum LA and LAR were recorded under controlled conditions. The foliar application of 1.0 PPM putrescine produced 39% more LA, 30% SLA, and 26% more LAR compared with controlled treatments (**Figure 1**).

### Effect of putrescine on plant height, tillers per plant, and biomass accumulation of wheat genotypes under drought stress

**Figure 2:**
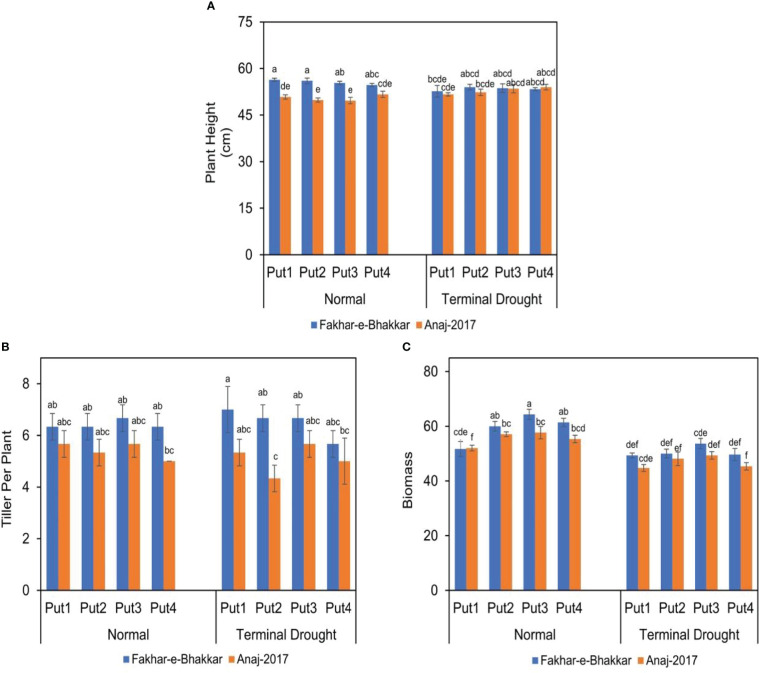
The effect of putrescine on the plant height **(A)**, tiller per plant **(B)**, and biomass (g plant-1) **(C)** of two wheat cultivars under normal and terminal drought conditions. Abbreviation: PPM, parts per million, Put1, putrescine 0 PPM (untreated control); Put2, putrescine 0.5 PPM; Put3, putrescine 1.0 PPM; Put4, putrescine 1.5 PPM. The different letters above the bar columns indicate significant differences in mean ± SE at p< 0.05. At least three independent biological replications were considered for each treatment.

The plant height of both genotypes was significantly affected under both normal and drought conditions (**Figure 2**). Among wheat cultivars, V1 attained significantly greater plant height, tillers per plant, and biomass accumulation than cultivar V2, under both normal and terminal stress conditions. Although putrescine improved the performance of both genotypes, especially under drought conditions, genotype V1 was more responsive to Putrescine than V2. Regarding plant height, putrescine application under all levels produced greater plant height than controlled treatments. Regarding the biomass per plant under all putrescine treatments, the 1.0 PPM was statistically superior to other concentrations, especially under drought conditions.

### Effect of putrescine on spike length, spike weight, and spikelet per spike of wheat genotypes under drought stress

**Figure 3:**
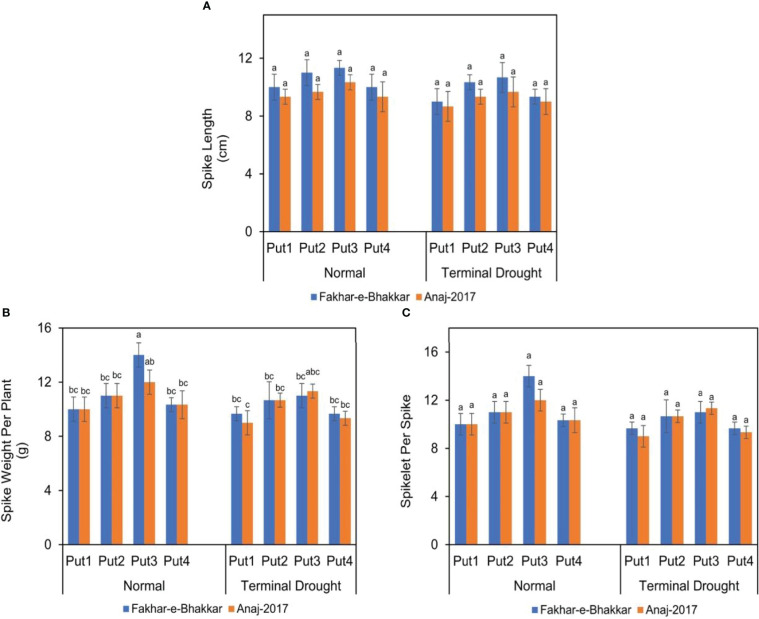
The effect of putrescine on the spike length **(A)**, spike weight **(B)**, and spikelets per spike **(C)** of two wheat cultivars under normal and terminal drought conditions. Abbreviation: PPM, parts per million, Put1, putrescine 0 PPM (untreated control); Put2, putrescine 0.5 PPM; Put3, putrescine 1.0 PPM; Put4, putrescine 1.5 PPM. The different letters above the bar columns indicate significant differences in mean ± SE at p< 0.05. At least three independent biological replications were considered for each treatment.

Wheat genotype Fakhar-e-Bhakkar (V1) showed a larger and heavier spike than the Anaj-2017 (V2), particularly under drought conditions (**Figure 3**), which suggests that V2 is more sensitive to terminal drought. The application of putrescine, particularly at 1.00 PPM, significantly reduced drought impact, which was more obvious in V1. The interaction between drought stress, genotypes, and putrescine application showed an insignificant effect on spike length and spikelets per spike (**Figure 3**), while it was found to significantly affect spike weight per plant. Under terminal drought stress, significantly, the spike weight per plant was observed with the foliar application of 1.0 PPM putrescine compared with other treatments (**Figure 3**).

### Effect of putrescine on MSI and RWC of wheat genotypes under drought stress

**Figure 4:**
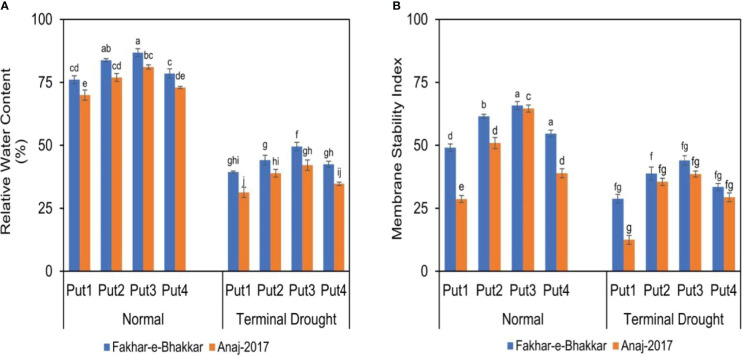
The effect of putrescine on the relative water content **(A)** and membrane stability index (%) **(B)** of two wheat cultivars under normal and terminal drought conditions. Abbreviation: PPM, parts per million, Put1, putrescine 0 PPM (untreated control); Put2, putrescine 0.5 PPM; Put3, putrescine 1.0 PPM; Put4, putrescine 1.5 PPM. The different letters above the bar columns indicate significant differences in mean ± SE at p< 0.05. At least three independent biological replications were considered for each treatment.

The figure shows that higher RWC and MSI were recorded in Fakhar-e-Bhakkar under well-watered conditions with the foliar application of 1.0 PPM putrescine compared with other treatments. However, considering the drought treatments, the foliar application of 1.0 PPM putrescine maintained higher RWC and MSI in Fakhar-e-Bhakkar compared with other combinations, while less RWC and MSI was observed in Anaj2017 with water spray under terminal drought stress (**Figure 4**).

### Effect of putrescine on grain yield of wheat genotypes under drought stress

**Figure 5:**
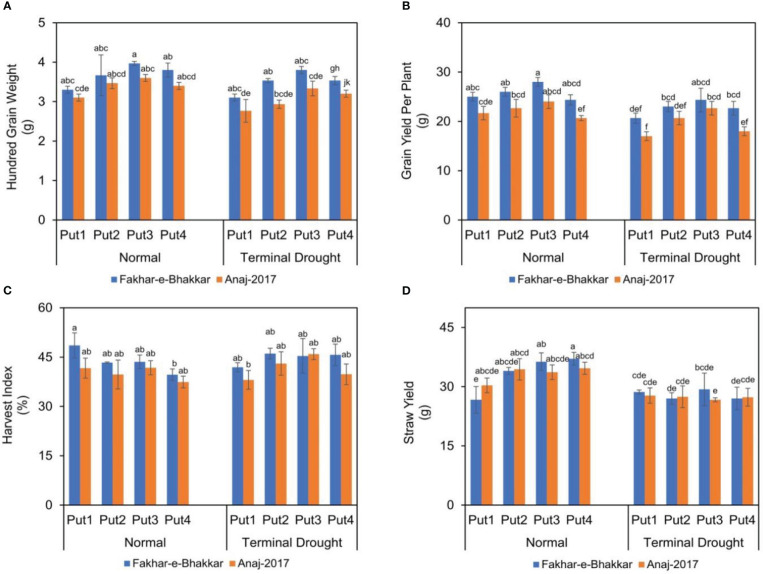
The effect of putrescine on the 100-grain weight **(A)**, grain yield **(B)**, harvest index **(C)**, and straw yield **(D)** of two wheat cultivars under normal and terminal drought conditions. Abbreviation: PPM, parts per million, Put1, putrescine 0 PPM (untreated control); Put2, putrescine 0.5 PPM; Put3, putrescine 1.0 PPM; Put4, putrescine 1.5 PPM. The different letters above the bar columns indicate significant differences in mean ± SE at p< 0.05. At least three independent biological replications were considered for each treatment.

Drought stress significantly reduced the grain yield and 100-grain weight in both genotypes (**Figure 5**). Statistically, a higher 100-grain weight was recorded in Fakhar-e-Bhakkar under both well-watered and terminal drought stress with the foliar application of putrescine. However, a higher grain yield was recorded under well-watered conditions with putrescine application. Regarding terminal drought stress, Fakhar-e-Bhakkar produced a higher grain yield per plant with the foliar application of 1.0 PPM putrescine compared with other treatments. Similarly, a higher straw yield was recorded in Fakhar-e-Bhakkar with the foliar application of 1.0 PPM putrescine compared with other combinations. A higher harvest index was observed in Fakhar-e-Bhakkar under well-watered conditions, which was statistically on par with all other combinations (**Figure 5**).

## Discussion

Climate change has worsened the outbreak of drought stress, particularly in the later reproductive stages of wheat. Therefore, the evaluation of different strategies to mitigate drought stress is necessary to enable wheat genotypes to perform well under drought conditions. This study investigates the drought tolerance and response of two wheat cultivars against putrescine application. The overall results of our study show that the wheat cultivar Fakhar-e-Bhakkar performed better against drought stress than Anaj-2017. In addition, in both genotypes, among four treatments of putrescine; the application of 1.0 PPM was found most effective against drought stress (**Figure 1**).

### Putrescine application improved the leaf area and leaf area ratio in wheat

Under drought stress, plants experience numerous morphological changes, such as reduced leaf size, leaf rolling, leaf senescence, and limited leaf extension, which could be the reason for lower LAR (leaf area ratio), LA (leaf area), and SLA in drought conditions. As Basu et al. (2016) reported drought stress could be the silent cause of leaf area reduction due to leaf rolling that minimizes water loss and decreases the activity of photosynthesis and the accumulation of dry matter. Reduced leaf area under drought stress might be due to reduced cell turgidity leading to a reduction in cell elongation, an imbalanced antioxidant defense system, and a higher number of reactive oxygen species (ROS) (Alishah et al., 2006; DaCosta and Huang, 2007; Zahoor et al., 2020). Similarly, Put improved leaf area, LAR, and SLA in the present study due to the regulation of LAI, turgidity, and photosynthetic activity (Farooq et al., 2009b).

### Putrescine application helped the wheat to retain higher biomass under drought stress

The current study shows that drought stress had no significant effect on plant height and the number of tillers per plant in wheat, as the timing and length of drought stress execution are important factors affecting plant height and tiller number. Drought stress at the heading stage had little influence on plant height due to the cessation of stem elongation and vegetative growth. Wheat tillers cease to grow between the commencement of stem elongation and anthesis. Drought stress during the grain- filling stage in wheat reduces plant height by up to 7% (Caverzan et al., 2016a). Many researchers have found that water stress causes a considerable reduction in plant growth, which can be reflected in leaf area, dry weight, and other important growth parameters and functions (Kilic and Yağbasanlar, 2010; Budak et al., 2013; El Sabagh et al., 2019). Similarly, drought stress significantly reduced the biomass and tillers in wheat genotypes in this study, but the 0.5-1.0 PPM application of putrescine mitigated the drought effects. In addition, the higher number of tillers in the Fakhr-e-Bhakkar genotype suggests that it has higher drought tolerance (**Figure 2**).

### Putrescine application improved the spike length and number of spikelets under drought stress

Data analysis unveiled that drought and putrescine both considerably affect grain yield. Drought execution in wheat genotypes at the heading stage reduced the number of spikelets per plant, causing a reduction in yield. Exogenous putrescine application of 1.0 PPM recorded a positive effect on spikelets per spike and the spike weight when compared with the control treatment (**Figure 3**) because of improved and higher RWC, MSI, and spikelet numbers. Scientists declared that grain yield was boosted due to increased spikelet numbers per spike (Philipp et al., 2018; Sakuma and Schnurbusch, 2020). Similarly, increased grain yield under putrescine application might be due to an improvement in growth and yield components as putrescine is involved in the production of abscisic acid (ABA) as well as osmotic adjustment and free radicals under abiotic stress (Velikova et al., 2000; Al-Kandari et al., 2009; Alcázar et al., 2010).

### Putrescine application reduced the oxidative damage caused by drought stress in wheat genotypes

Data analysis of RWC and MSI revealed that drought-stressed plants have a lower water content than well-watered plants. Drought stress enforces physiological changes in the integrity of plant cell membranes. Consistent with Awasthi et al. (2014), drought stress boosts electrolyte leakage and enhances the instability of the membrane, together with lessening the chlorophyll content, having a huge impact on RWC. According to Reddy et al. (2004), drought stress has an inverse relationship with the water content of plant tissue; an increase in the severity and duration of drought stress results in reduced water content. Our investigation results of RWC and MSI also show that prolonged drought conditions diminish the membrane stability index and RWC. Alternatively, the exogenous putrescine application at the heading stage under terminal drought stress helped to retain the RWC inside the plant and reduced electrolyte leakage and ROS in contrast with no putrescine application. Putrescine, being a scavenger of “free radicals” or ROS detoxifiers, protected the membrane from oxidative injury. There is strong evidence to suggest that polyamines (PAs), like putrescine, have the ability to eliminate ROS by binding the conjugates directly (Hussain et al., 2011). Putrescine application within the 0.5-1.0 PPM range under drought stress alleviated the cell membrane injury in both wheat genotypes (**Figure 4**).

Foliar putrescine application upgraded the status of RWC (relative water content) under drought stress in wheat. It enhanced the organic compatible solute accretion that helps to avoid water loss in plants by increasing solute potential (Alcázar et al., 2010). The improvement in RWC through the foliar application of putrescine might be due to ROS’s scavenging role and ability to regulate osmotic balance (Al-Kandari et al., 2009; Alcázar et al., 2010). Exogenous application of putrescine improved MSI under drought stress. This might be due to the regulation of membrane integrity and antioxidant activity mediated by the translation of specific genes, which helps plants to resist ROS produced under drought stress (Bajguz, 2000; Takahashi and Kakehi, 2009).

### Putrescine application helps to retain the yield and harvest index of wheat under drought stress

Data analysis demonstrated that water stress imposes the most prominent adverse effects in the Anaj-2017 genotype. Drought stress at the heading stage influenced the yield of terminal stress-sensitive wheat genotype and attributed to the severe reduction in yield components, namely biological yield, spikelet number, and grain weight. It was observed that drought stress reduced the yield of drought-sensitive wheat genotypes as a result of a decrease in photosynthetic parameters and chlorophyll content (Wasaya et al., 2021). Thus, it is obvious that stomata behavior, water status, and photosynthesis response to drought stress distinguish between sensitive genotypes and drought-tolerant genotypes (Munns et al., 2010). In relative terms, the foliar application of putrescine at the heading stage not only improves the yield of wheat genotypes but also assists in mitigating drought stress (**Figure 5**).

Data analysis showed that Fakhar-e-Bhakkar produced higher biological and grain yields than Anaj-2017 under drought stress, which could be attributed to the higher number of spikelets per spike, spike weight per plant relative to water content, and 100-grain weight. It was observed that some genotypes are able to complete the life cycle under moisture stress and maintain their yield compared with drought-susceptible genotypes due to their genetic makeup (Ahmad et al., 2009). Grain weight and spikelet number are the most effective and essential variables that could influence the grain yield of the wheat genotype (Leilah and Alkhateeb, 2005). Drought stress execution in the reproductive phase affects the traits contributing to yield parameters (Bayoumi et al., 2008). Fakhar-e-Bhakkar is a drought- resistant wheat genotype and attained the highest plant height, biomass, number of tillers, spike weight, number of spikelets per spike, grain weight per spike, 100-grain weight, and grain yield under drought stress and well-watered conditions. Because of its prodigious genetic makeup and its enzymatic defense system and osmolyte accumulation, the adverse effects of drought stress in the Fakhar-e-Bhakkar genotype are mitigated. Meanwhile, the wheat genotype Anaj-2017 recorded the lowest grain yield, being drought-sensitive, because sink capacity for dry matter accumulation is susceptible to drought conditions; nonetheless, this may be responsible for its low yield (Bayoumi et al., 2008).

## Conclusion

The current study recommends that the foliar application of polyamine, such as putrescine, is the best approach to tackle and mitigate undesirable drought effects in wheat. A higher grain yield was achieved under the putrescine application, probably due to improved spike weight, spikelet numbers, and 100-grain weight, particularly in the stress-tolerant genotype (Fakhar-e-Bhakkar). According to the research findings, the foliar application of putrescine improved the membrane stability index and relative water content, which results in enhanced 100-grain weight and spike weight. Wheat cultivar Fakhar-e-Bhakkar showed more tolerance against terminal drought than Anaj-2017. Similarly, Fakhar-e-Bhakkar was most responsive to the putrescine application, and the foliar application of putrescine (1.0 PPM) has the potential to stabilize wheat against terminal drought stress. Our study suggests that Fakhar-e-Bhakkar has the potential to grow and survive in low- water and well-watered conditions, so farmers can grow this wheat genotype for longer with better outcomes.

## Data availability statement

The datasets presented in this study can be found in online repositories. The names of the repository/repositories and accession number(s) can be found in the article/supplementary material.

## Author contributions

AW and TY: conceptualization. IR: data collection. AW, IR and TY: writing the original draft. AD, MK, MJ, GR, IA, ME-H, MB, MR, and AS: review and editing. All authors contributed to the article and approved the submitted version.
