# Corrigendum: Foliar application of putrescine alleviates terminal drought stress by modulating water status, membrane stability, and yield- related traits in wheat (*Triticum aestivum* L.)

**DOI:** 10.3389/fpls.2023.1231723

**Published:** 2023-06-27

**Authors:** Allah Wasaya, Iqra Rehman, Atta Mohi Ud Din, Muhammad Hayder Bin Khalid, Tauqeer Ahmad Yasir, Muhammad Mansoor Javaid, Mohamed El-Hefnawy, Marian Brestic, Md Atikur Rahman, Ayman El Sabagh

**Affiliations:** ^1^ Department of Agronomy, Bahauddin Zakariya University Multan, Multan, Pakistan; ^2^ College of Agriculture, University of Layyah, Layyah, Pakistan; ^3^ National Research Center of Intercropping, The Islamia University of Bahawalpur, Multan, Pakistan; ^4^ Department of Agronomy, College of Agriculture, University of Sargodha, Sargodha, Pakistan; ^5^ Department of Chemistry, Rabigh College of Sciences and Arts, King Abdulaziz University, Jeddah, Saudi Arabia; ^6^ Department of Plant Physiology, Slovak University of Agriculture, Nitra, Slovakia; ^7^ Grassland and Forage Division, National Institute of Animal Science, Rural Development Administration, Cheonan, Republic of Korea; ^8^ Department of Agronomy, Faculty of Agriculture, Kafrelsheikh University, Kafr al-Sheik, Egypt; ^9^ Department of Field Crops, Faculty of Agriculture, Siirt University, Siirt, Türkiye

**Keywords:** bread wheat, yield, terminal drought, putrescine, leaf area ratio, membrane stability index

Incorrect Acknowledgments

In the published article, there was an error in the Acknowledgments statement.

The authors gratefully acknowledge technical and financial support provided the Ministry of Education and King Abdulaziz University, DSR, Jeddah, Saudi Arabia for funding this research work to publish through the project number (IFPIP:1166-662-1443). The authors extend their appreciation to VEGA1/0664/22 Mitigation of the effects of environmental stresses in photosynthesis and plant production.

The correct Acknowledgments statement appears as follows.

Acknowledgments

The authors gratefully acknowledge the technical and financial support provided by the Ministry of Education and King Abdulaziz University, DSR, Jeddah, Saudi Arabia, to this research work through project number IFPIP: 1166-662-1443.

The authors apologize for this error and state that this does not change the scientific conclusions of the article in any way. The original article has been updated.

